# Impending Backlog of Cleft Palate Patients Due to COVID-19

**DOI:** 10.5334/aogh.3534

**Published:** 2022-01-20

**Authors:** Jacob J. Wood, Garrick Gu, Robert D. Guber, Douglas M. Rothkopf

**Affiliations:** 1 Division of Plastic Surgery, University of Massachusetts Chan Medical School, Worcester, Massachusetts, US; 2 Division of General Surgery, University of Massachusetts Chan Medical School, Worcester, Massachusetts, US

## Abstract

Coronavirus disease 2019 (COVID-19) has placed an unprecedented strain on healthcare systems worldwide, but while high-income countries (HICs) have been able to adapt, low- and middle-income countries (LMICs) have been much slower to do so due to a lack of funding, skilled healthcare providers, equipment, and facilities. The redistribution of resources to combat the pandemic in LMICs has resulted in decreased surgical volumes at local surgical centers as well as a dramatic reduction in the number of humanitarian aid missions. Despite recent global investment in improving the surgical capacities of LMICs, even in the pre-COVID-19 era there was a vast unmet surgical need. This deficit in surgical capacity has grown during the pandemic and it will be a significant struggle to overcome the resulting backlog of patients. A topic of particular concern to the authors is the effect that the pandemic will have on the delivery of time-sensitive surgical care to patients with cleft palate deformities as delay in providing care can have enormous physical and psychosocial consequences. This paper draws increased attention to the lasting impact that the COVID-19 pandemic may have on cleft palate patients in LMICs.

**SSRN Pre-print server link:** *https://papers.ssrn.com/sol3/papers.cfm?abstract_id=3898055*

Humanitarian mission trips have historically played a significant role in providing surgical care to patients in LMICs. In recent years, investments in global health have shifted away from humanitarian mission trips to increasing the capacity of local healthcare facilities and training local surgeons [1]. However, humanitarian trips of this kind are still responsible for performing nearly 250,000 operations each year [2]. The travel restrictions imposed by many countries to stem the spread of COVID-19 curbed these trips. One such example is Operation Smile, which cancelled all direct patient care at the beginning of the pandemic and is now slowly restarting. They report that they have over 10,000 patients awaiting treatment [3]. One recent article using data from Smile Train found that there was a 31% reduction in procedures to correct cleft palate between 2019 and 2020 [4]. With the arrival of the COVID-19 vaccines, humanitarian organizations should be able to increase the volume of cleft palate procedures performed. However, challenges will arise, such as surgeons’ inability to travel due to safety concerns or pandemic-associated case build-up at their home institutions.

In LMICs, local surgeons and hospitals are struggling to maintain their pre-COVID surgical volume. A study published by the World Health Organization determined that while HICs have been able to recover much of their surgical volume as of August 2020 after an initial decline in March 2020, the surgical volume in LMIC’s has remained suppressed [5]. This prolonged reduction in surgical capacity in LMICs as a result of COVID-19 is producing a backlog that will be extremely challenging to overcome. The COVIDSURG Collaborative estimates that 28,404,603 surgical cases were cancelled worldwide over a 12-week period during the pandemic, and that if pre-pandemic surgical volume was increased by 20%, it would take 45 weeks to recover from this backlog [6]. LMICs lack the same resources possessed by HICs, creating a risk that this backlog will never be reduced without external support. Further confounding this backlog, it is estimated that the cancellation of elective surgeries in the US resulted in loss of approximately $4 to $5.4 billion in revenue each month [7]. This further strain of revenue might limit mission trips as hospitals first seek to recoup their losses. The pandemic has lasted for longer than 12 weeks and is likely to continue affecting LMICs long after HICs have returned to normal with the aid of vaccination campaigns. Despite representing only 14% of the global population, HICs have secured 51% of the current COVID-19 vaccine supply [8].

Treatment of patients with cleft palate during and after the pandemic is of great concern due to the time-sensitive nature of the procedure, and care should not be interrupted due to the pandemic [9]. A study by Breugem et al. surveyed providers from the 2015 European Cleft Craniofacial Meeting, and 92% of providers suggested that cleft palate repair should be performed by 18 months of age [10]. When treatment of cleft palate is delayed there can be a host of destructive physical and psychosocial consequences. Infants born with cleft palate can have difficulty breastfeeding, leading to nutritional insufficiency and reduced growth and development [11]. Additionally, people with untreated cleft palate can suffer from nasal regurgitation, leading to possible lasting pain and discomfort [12]. Socially, untreated cleft palate poses several challenges. People left untreated may lack the ability to communicate effectively with strangers or even family members due to an inability to accurately articulate speech sounds [12,13]. Social isolation is also a consequence, particularly in communities where there has been a lack of education about cleft palate. Research has also shown that people with untreated cleft palate were more likely to report bullying from peers and to drop out of school [12]. Children with untreated cleft palate have significantly increased rates of social anxiety and major depressive disorder [14]. The inability for people with cleft palate to fully participate in society is devastating on an individual, familial, and societal level. The unemployment or underemployment of individuals with untreated cleft palate contributes a significant loss to their country’s GDP and negatively affects their ability to provide for themselves and their families [15].

We recommend that now is the time to take proactive steps to mitigate the consequences that this pandemic will have for children with cleft palate and to investigate solutions to reduce the impact the next pandemic or disaster will have on global health. Organizations previously engaged in humanitarian mission trips should consider reallocating their funding to local hospitals in LMICs, with the goals of training physicians, providing surgical and medical care domestically, and developing sustainable systems. An estimate made in 2016 placed the annual expenditures on humanitarian mission trips at $3.7 billion from the United States alone [16]. In 2019, the average healthcare spending per capita among countries designated as lower-middle income and low income by the World Bank was $81 and $40 respectively [17], which underlines how significant an investment of potentially $3.7 billion could be to these populations. Additional investments could be made in telemedicine and interpreter services to reduce the burden on local physicians in LMICs and to provide opportunities to volunteer in global health in the absence of humanitarian mission trips. In addition to financial investments, a commitment must be made to distribute COVID-19 vaccines equitably across the world. As of December 8, 2021, vaccination rates in lower-middle income and lower-income countries are 26.36% and 3.32% respectively compared to 64.54% in high-income countries [18]. Until there is parity in access to COVID-19 vaccines worldwide, the recovery of the healthcare systems in LMICs may be suppressed.

It is vitally important that governments in LMICs increase their domestic healthcare expenditures to develop health systems better equipped to care for their populations in the absence of foreign aid and mission trips. The COVID-19 pandemic has painfully illustrated how fragile a system relying on care delivered by foreign physicians can be. Investments made by all players in global health should not be limited only towards recovery from the current crisis of COVID-19, but also be directed towards preparing for the next crisis by creating more robust and sustainable systems.

Any effort to help LMICs in the wake of COVID-19 to recover their surgical volume should be highly coordinated, especially since global resources will already be stretched thin. While the backlog in cleft palate surgical care may not be the top priority with the pandemic still raging around the world, it should be under consideration and planning as delaying the treatment of these children with cleft palates can lead to a lifetime of physical, social, and economic disability.

## References

[1] Meara JG, Greenberg SLM. The Lancet Commission on Global Surgery 2030: Evidence and solutions for achieving health, welfare and economic development. Surgery. 2015; 157(5): 834–835. DOI: 10.1016/j.surg.2015.02.009 25934019

[2] Medoff S, Freed J. The need for formal surgical global health programs and improved mission trip coordination. Annals of Global Health. 2016; 82(4): 634. DOI: 10.1016/j.aogh.2016.08.003 27986232

[3] Hope on the horizon: Safely resuming surgery and care. Operation Smile. https://www.operationsmile.org/blog/hope-horizon-safely-resuming-surgery-and-care. Accessed July 12, 2021.

[4] Vander Burg R, Agrawal K, Desai P, Desalu I, Donkor P. Impact of COVID-19 on elective cleft surgery in low- and middle-income countries. Plastic and Reconstructive Surgery – Global Open. 2021; 9(6): pe3656. DOI: 10.1097/GOX.0000000000003656 PMC8219249 34168945

[5] O’Reilly-Shah VN, Van Cleve W, Long DR, et al. Impact of COVID-19 response on global surgical volumes: an ongoing observational study. Bulletin of the World Health Organization. 2020; 98(10): 671–682. DOI: 10.2471/BLT.20.264044 33177757 PMC7652560

[6] COVIDSURG Collaborative. Elective surgery cancellations due to the COVID-19 pandemic: Global predictive modelling to inform surgical recovery plans. British Journal of Surgery; 2020; 107(11): 1440–1449. DOI: 10.1002/bjs.11746 PMC7272903 32395848

[7] Best MJ, McFarland EG, Anderson GF, Srikumaran U. The likely economic impact of fewer elective surgical procedures on US hospitals during the COVID-19 pandemic. Surgery. 2020; 168(5): 962–967. DOI: 10.1016/j.surg.2020.07.014 32861440 PMC7388821

[8] So AD, Woo J. Reserving coronavirus disease 2019 vaccines for global access: Cross-sectional analysis. BMJ. 2020; m4750. DOI: 10.1136/bmj.m4750 33323376 PMC7735431

[9] Alexandre LP, Cançado LN, Pretti H, et al. Importance of the treatment of patients with lip and palate cleft, especially during the COVID-19 pandemic. Oral Surgery; 2020; 10.1111/ors.12593. DOI: 10.1111/ors.12593 PMC7753365 33362877

[10] Breugem C, Smit H, Mark H, et al. Prioritizing cleft/craniofacial surgical care after the COVID-19 pandemic. Plastic and Reconstructive Surgery – Global Open. 2020; 8(9): e3080. DOI: 10.1097/GOX.0000000000003080 33133937 PMC7544383

[11] Martin V, Greatrex-White S. An evaluation of factors influencing feeding in babies with a cleft palate with and without a cleft lip. Journal of Child Health Care. 2013; 18(1): 72–83. DOI: 10.1177/1367493512473853 23439590

[12] Rees J, Muskett T, Enderby P, Stackhouse J. Implications of untreated cleft palate in the developing world: Adaptation of an outcome measure. Folia Phoniatrica et Logopaedica. 2016; 68(1): 1–9. DOI: 10.1159/000440836 27362363

[13] Bruneel L, Luyten A, Bettens K, et al. Delayed primary palatal closure in resource-poor countries: Speech results in Ugandan older children and young adults with cleft (lip and) palate. Journal of Communication Disorders. 2017; 69: 1–14. DOI: 10.1016/j.jcomdis.2017.06.010 28675808

[14] Demir T, Karacetin G, Baghaki S, Aydin Y. Psychiatric assessment of children with nonsyndromic cleft lip and palate. General Hospital Psychiatry. 2011; 33(6): 594–603. DOI: 10.1016/j.genhosppsych.2011.06.006 21816483

[15] Muntz HR, Meier JD. The financial impact of unrepaired cleft lip and palate in the Philippines. International Journal of Pediatric Otorhinolaryngology. 2013; 77(12): 1925–1928. DOI: 10.1016/j.ijporl.2013.08.023 24139590

[16] Caldron PH, Impens A, Pavlova M, Groot W. Economic assessment of US physician participation in short-term medical missions. Global Health. 2016; 12(1): 45. Published 2016 Aug 22. DOI: 10.1186/s12992-016-0183-7 27549787 PMC4994162

[17] Global Burden of Disease Health Financing Collaborator Network. Past, present, and future of global health financing: A review of development assistance, government, out-of-pocket, and other private spending on health for 195 countries, 1995–2050. Lancet. 2019 Jun 1; 393(10187): 2233–2260. Epub 2019 Apr 25. Erratum in: *Lancet*. 2021 Sep 11; 398(10304): 956. PMID: 31030984; PMCID: PMC6548764. DOI: 10.1016/S0140-6736(19)30841-4 31030984 PMC6548764

[18] WHO Coronavirus (COVID-19) Dashboard. World Health Organization. https://covid19.who.int/table. Accessed December 8, 2021.

